# Comparative Transcriptomic Exploration Reveals Unique Molecular Adaptations of Neuropathogenic *Trichobilharzia* to Invade and Parasitize Its Avian Definitive Host

**DOI:** 10.1371/journal.pntd.0004406

**Published:** 2016-02-10

**Authors:** Roman Leontovyč, Neil D. Young, Pasi K. Korhonen, Ross S. Hall, Patrick Tan, Libor Mikeš, Martin Kašný, Petr Horák, Robin B. Gasser

**Affiliations:** 1 Department of Parasitology, Faculty of Science, Charles University in Prague, Prague, Czech Republic; 2 Faculty of Veterinary and Agricultural Sciences, The University of Melbourne, Melbourne, Victoria, Australia; 3 Genome Institute of Singapore, Singapore, Republic of Singapore; 4 Cancer and Stem Cell Biology, Duke-NUS Graduate Medical School, Singapore, Republic of Singapore; 5 Department of Botany and Zoology, Faculty of Science, Masaryk University, Brno, Czech Republic; George Washington University School of Medicine and Health Sciences, UNITED STATES

## Abstract

To date, most molecular investigations of schistosomatids have focused principally on blood flukes (schistosomes) of humans. Despite the clinical importance of cercarial dermatitis in humans caused by *Trichobilharzia regenti* and the serious neuropathologic disease that this parasite causes in its permissive avian hosts and accidental mammalian hosts, almost nothing is known about the molecular aspects of how this fluke invades its hosts, migrates in host tissues and how it interacts with its hosts’ immune system. Here, we explored selected aspects using a transcriptomic-bioinformatic approach. To do this, we sequenced, assembled and annotated the transcriptome representing two consecutive life stages (cercariae and schistosomula) of *T*. *regenti* involved in the first phases of infection of the avian host. We identified key biological and metabolic pathways specific to each of these two developmental stages and also undertook comparative analyses using data available for taxonomically related blood flukes of the genus *Schistosoma*. Detailed comparative analyses revealed the unique involvement of carbohydrate metabolism, translation and amino acid metabolism, and calcium in *T*. *regenti* cercariae during their invasion and in growth and development, as well as the roles of cell adhesion molecules, microaerobic metabolism (citrate cycle and oxidative phosphorylation), peptidases (cathepsins) and other histolytic and lysozomal proteins in schistosomula during their particular migration in neural tissues of the avian host. In conclusion, the present transcriptomic exploration provides new and significant insights into the molecular biology of *T*. *regenti*, which should underpin future genomic and proteomic investigations of *T*. *regenti* and, importantly, provides a useful starting point for a range of comparative studies of schistosomatids and other trematodes.

## Author Summary

Despite the clinical importance of *Trichobilharzia regenti* in bird hosts and as a cause of cercarial dermatitis in humans, almost nothing is known about the molecular aspects of this fluke and its interactions with its hosts. Here, we sequenced, assembled and annotated the transcriptome representing two life stages (cercariae and schistosomula) of *T*. *regenti* involved in the first phases of infection of the bird host. We identified key biological and metabolic pathways specific to each of these two developmental stages and also undertook comparative analyses using data available for related flukes. Detailed analyses showed the unique involvement of carbohydrate metabolism, translation and amino acid metabolism, and calcium in *T*. *regenti* cercariae during invasion and in growth and development, as well as cell adhesion molecules, microaerobic metabolism (citrate cycle and oxidative phosphorylation), peptidases (cathepsins) and other histolytic and lysozomal proteins in schistosomula during migration in neural tissues. These molecular insights into *T*. *regenti* biology should support future genomic and proteomic investigations of *T*. *regenti*, and comparative studies of flatworms.

## Introduction

The bird fluke *Trichobilharzia regenti* is a member of the Schistosomatidae (= blood flukes; Class Trematoda), a family of parasitic flatworms of medical and veterinary importance [1,2]. *T*. *regenti* is widely distributed geographically and is highly prevalent, for instance, in parts of Europe (including Russia), New Zealand and Iran [3–6]. Like blood flukes of the genus *Schistosoma*, *T*. *regenti* is dioecious, has a two-host life cycle (including a lymnaeid snail of the genus *Radix*) and has an invasive furcocercarial stage that actively penetrates the skin of a definitive vertebrate host. Unlike members of the genus *Schistosoma*, *T*. *regenti* invades and migrates through skin and nerves to then establish within the nasal mucosa [7–9]. During its aquatic phase, *T*. *regenti* can accidently penetrate human skin and cause cercarial dermatitis. Cercarial dermatitis, caused by avian schistosomes, is regarded as an emerging disease [10–12] although global economic losses are not known, it is accepted that this condition can have a considerable impact on local, tourism-based economies, and may also represent a debilitating occupational disease of rice farmers (see Horák et al., 2015 for review [12]). As avian (including *T*. *regenti*) and human schistosomes can occur in the same water reservoirs, there are at least two issues of relevance in relation to the differential diagnosis of disease: (a) Based on clinical signs, cercarial dermatitis caused by avian schistosomes can be confused with that caused by human schistosomes [13]. (b) Prevalence surveys of hepatointestinal or urogenital schistosomiasis of humans might be influenced/affected by serological cross-reactivity resulting from exposure to cercariae of avian schistosomes [14].

To better understand *T*. *regenti* and the diseases that this parasite causes, considerable research has focused on exploring its life cycle. Once shed from the intermediate aquatic snail host, the cercariae survive only for a limited time in water (1 to 1.5 days in related avian schistosomes [15], consuming their glycogen reserves acquired from the intermediate host [16]. Upon contact with the skin of the definitive host, the cercariae release secretions containing proteolytic enzymes (peptidases) from their circumacetabular and postacetabular penetration glands [17], which enable tissue penetration [18–20]. During penetration, the cercariae transform to schistosomula within ~ 12 h [9,21]; for schistosomes, this process is accompanied by a loss of their tail, formation of a double (heptalaminar) membrane covering the tegument and a reduction of surface glycocalyx [21,22] as well as a switch from aerobic to anaerobic metabolism, depending on the amount of accessible glucose [23,24] and the activation of metabolic processes in the parasite’s gut [25]. In contrast to human schistosomes, *T*. *regenti* schistosomula do not migrate directly to blood vessels, but rather enter peripheral nerves, and migrate to the spinal cord and brain of the host, during which they feed on neural tissue [8,26]. Having reached the pre-adult stage in the meninges, the schistosomula start to feed on blood and then migrate into the nasal cavity, likely via an intravascular route [27]. The significant damage to nerve tissue caused by migrating schistosomula can lead to behavioural changes, disorientation, paralysis or even death in some hosts [7,28].

Despite the importance of cercarial dermatitis in humans caused by *T*. *regenti* and the unique neuropathogenic effects of this parasite on its permissive avian hosts as well as experimental rodent hosts, little is known about the molecular mechanisms underlying tissue penetration, transformation of cercariae to schistosomula, tissue invasion and parasite-host interactions. Here, we propose that exploring the developmental transcriptomes of cercaria and schistosomulum of *T*. *regenti* will provide vital insights into the fundamental molecular biology of this parasite, and identify essential pathways and protein classes linked to early tissue invasion. An analysis of the developmental transcriptome of *T*. *regenti* should also fill gaps in our knowledge of the parasite’s biology, as, to date, major molecular investigations have focused mainly on human blood flukes. Therefore, in the present study, we (i) assembled and annotated the transcriptome of two consecutive life stages of *T*. *regenti* involved in the first phases of infection of the avian host, (ii) identified key biological and metabolic pathways specific to each of these two developmental stages, and (iii) undertook comparative analyses using data available for taxonomically related blood flukes of the genus *Schistosoma*.

## Materials and Methods

### Ethics statement

The maintenance and care of experimental animals was carried out in accordance with the European Directive 2010/63/EU and Czech law (246/1992 and 359/2012) for biomedical research involving animals. Experiments have been performed under legal consent of the Expert Commission of the Section of Biology, Faculty of Science, Charles University in Prague and the Ministry of Education, Youth and Sports of the Czech Republic (ref. no. MSMT-31114/2013-9).

### Parasite materials

**Fig 1:**
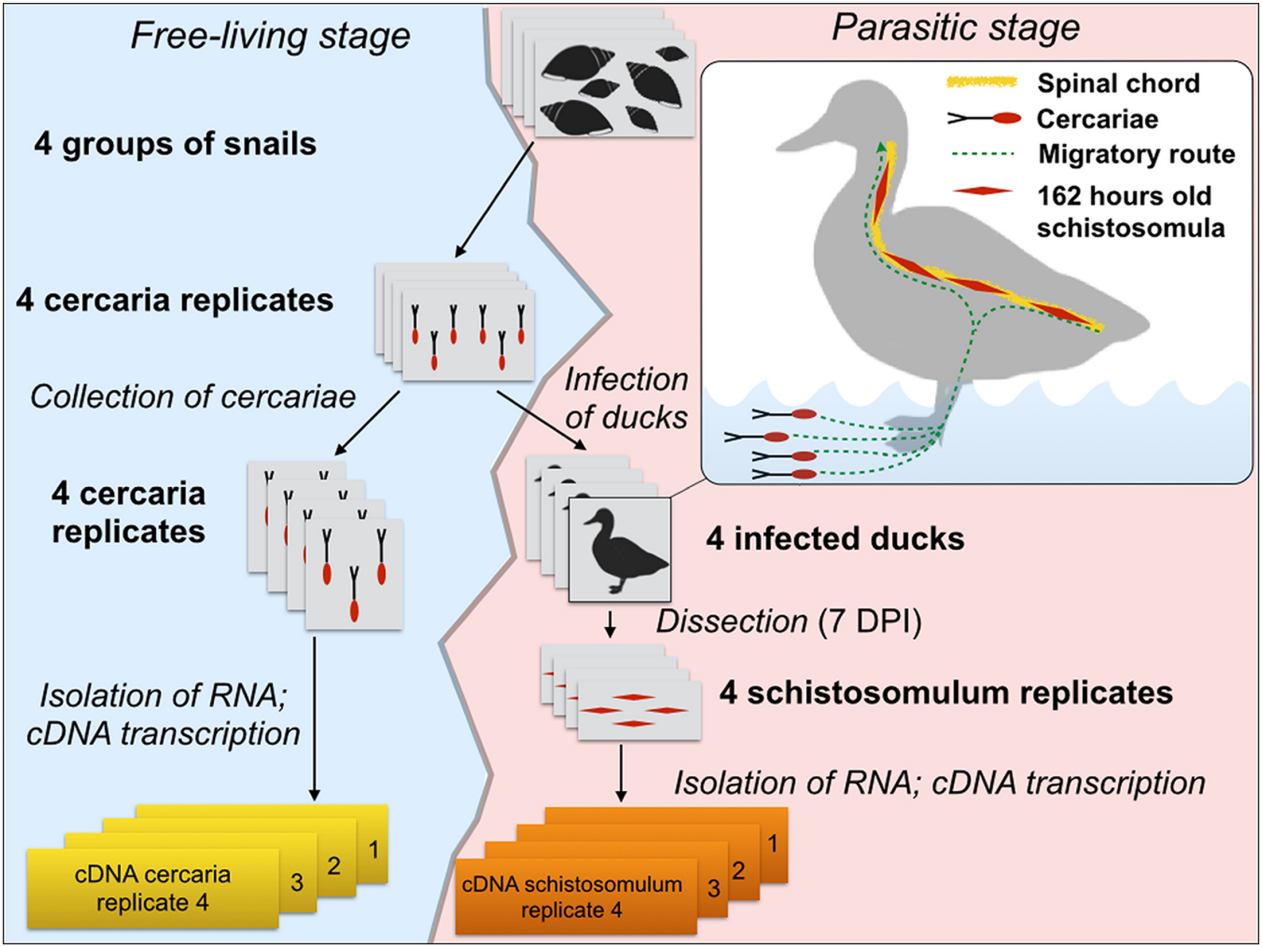
Experimental design for the production of *Trichobilharzia regenti* cercariae and schistosomula for the present study.

*Trichobilharzia regenti* was maintained in snail intermediate (*Radix lagotis*) and definitive (*Anas platyrhynchos* f. *domestica*; breed—Cherry Valley strain) hosts in the Laboratory of Helminthology, Faculty of Science, Charles University in Prague, using an established protocol [1]. Four separate groups of infected snails (*n* = 20 snails per group), each representing a distinct biological replicate, were established to obtain four independent biological replicates of pooled cercarial and schistosomula samples (Fig 1).

Schistosomulum replicates: Upon light stimulation for 2 h, cercariae (mixed-gender) shed from each snail group were collected and used to infect seven-day old ducks (*n* = 4 replicates; 2,500 cercariae per duck). Pooled schistosomula were collected from the spinal cord of each infected duckling seven days following inoculation using established methods [7,28]. Briefly, the spinal cord was carefully prised apart manually (using dissection needles) in phosphate-buffered saline (PBS) and exposed to bright light for 1 h. Schistosomula (*n* = 4 replicates; 150 to 400 individuals per duckling) were collected, washed extensively in PBS and stored in TRIzol reagent (Invitrogen) at -80°C until further processing. For each biological replicate, a sample of cercariae (shed from the same snail group used to infect the ducks) was also collected for RNA isolation. Cercaria replicates: Cercariae were collected every second day for one week, with each batch washed twice in tap water, centrifuged at 2,500 ×*g* at 4°C, and then stored in TRIzol reagent (Invitrogen) at -80°C.

### RNA isolation, library preparation and sequencing

Total RNA was purified from individual biological replicates (four for both cercariae and schistosomula) using TRIzol, and residual genomic DNA removed (DNA-free kit, Invitrogen) following the manufacturer’s instructions. The integrity and quality of total RNA were determined using a Bioanalyzer 2100 (Agilent) and Qubit RNA BR assay kit (Invitrogen). Messenger RNA (mRNA) was purified, and short-insert (330 bp) complementary DNA (cDNA) libraries constructed and barcoded according to the manufacturer’s instructions (TruSeq RNA Sample Preparation v.2, Illumina). All cDNA library was paired-end sequenced (2 × 211 base reads) on a single line using the HiSeq 2500 platform (Illumina).

### Assembly of the transcriptome

Sequencing adaptors and nucleotides with a Phred quality score of < 20 were removed using Trimmomatic v.0.3 [29]. The quality of filtered paired-end read data was manually assessed using FastQC [30] For each RNA-seq dataset, reads were corrected using Spades v.3.1.0 [31] and normalized digitally using the program khmer v.1.1 [32]. For each of the cercarial and schistosomula RNA-seq data sets, replicate datasets were pooled and used to assemble non-redundant transcriptomes using Oases v.0.2.8 [33], employing coverage cut-offs of 13 and 14, and *k*-mer values of 47 and 49, respectively. A final, merged transcriptome was generated by concatenating cercarial and schistosomula transcriptomes, and removing redundancy using CD-HIT-EST [34] using a nucleotide sequence identity threshold of 85%. Finally, coding domains were predicted using Transdecoder [35], and only transcripts encoding proteins of ≥ 30 amino acids were retained. The proportion of genome annotation represented by the non-redundant larval transcriptome was assessed using the program CEGMA [36].

### Annotation of the transcriptome

Transcripts homologous (BLASTn; E-value cut-off: < 10^−5^) to avian, bacterial or viral nucleotide sequences in the NCBI non-redundant nucleotide database [37] and translated proteins homologous (BLASTp; E-value cut-off: 10^−5^) to transposable elements in the RepBase database [38] were quarantined as well as sequences shorter than 50 amino acids (aa) with no homology in public databases. The non-redundant, merged transcriptome was then annotated using an established pipeline [39]. Briefly, protein sequences inferred from transcripts were annotated using their closest homologues (BLASTp; E-value cut-off: ≤ 10^−5^) in the following databases: NCBI non-redundant protein [37]; SwissProt [40]; MEROPS peptidase and peptidase inhibitor [41]—with predicted peptidases of unknown catalytic type and inhibitor homologues of unassigned peptidases being excluded; Kyoto Encyclopedia of Genes and Genomes (KEGG) [42], excluding KEGG “Human Diseases” and “Organismal Systems” categories. Conserved domains and their associated gene ontology (GO) annotations were predicted using the program InterProScan [43]. The server REVIGO was used to summarize GO terms and define a representative subset of terms using a simple clustering algorithm that relies on semantic similarity measures [44]. Excretory/secretory (E/S) proteins were predicted based on the presence of a signal peptide and the lack of a transmembrane domain; in addition, proteins were subjected to analysis using the program MultiLoc2 [45] to predict their sub-cellular location. Only proteins predicted to be extracellular or lysosomal (score: > 0.5) were included from the final set of predicted E/S proteins.

### Analysis of differential transcription

For each biological replicate, paired, trimmed and corrected reads were mapped to the final transcriptome using RSEM [46]. The expected counts predicted were rounded to the highest whole number, and used as counts per transcript for differential gene transcription analysis using edgeR v.3.6.7 [47] and R v.3.10 [48] software packages, with read counts being normalized to account for any GC bias [49] and using the trimmed mean of M-values (TMM) [50]. Transcripts with more than a log_2_ fold-change in transcription between the two developmental stages (i.e. cercaria and schistosomulum) and with a false discovery rate (FDR) of ≤ 0.01 were recorded as differentially transcribed. Enriched GO terms for transcripts recorded to be differentially transcribed in either developmental stage were tested using topGO [51] and the Fisher’s exact test (p ≤ 0.05); representative enriched GO terms were inferred using the program REVIGO [44]. In addition, transcripts specific to either the cercaria or the schistosomulum were those with RSEM expected counts of > 2 in at least one of four replicates representing one but not the other developmental stage.

## Results

### Characterisation and annotation of the non-redundant transcriptome of *T*. *regenti*

The sequencing of eight *T*. *regenti* cDNA libraries (four representing each the cercaria and schistosomulum biological replicates) produced 146,921,480 high quality reads (75,733,168 for cercariae; 71,188,312 for schistosomula), with an average read length of 132 ± 30 bp (mean ± standard deviation; Tables 1 and S1). Pooled RNA-seq data were used to assemble a non-redundant transcriptome, which included 12,705 assembled transcripts (average nucleotide length: 2,697.1 ± 2,166.4 bp; 115 to 41,111 bp; N50 = 3,333), each encoding a predicted protein (average length: 514.5 ± 537.7 residues; range: 30 to 8,133 residues), excluding transcripts that did not code for a protein (n = 6,899). Of the selected coding regions, 9,514 commenced with a start codon, and 11,434 terminated with a stop codon. Despite sequencing only two developmental stages, the *T*. *regenti* transcriptome includes 89.5% of the 358 conserved eukaryotic genes [36] (Table 1), and thus represents a substantial proportion of the gene set. On average, 61.5% and 74.4% of the sequence reads representing the cercaria and schistosomulum, respectively, mapped to the non-redundant *T*. *regenti* transcriptome (S1 Table). The final transcriptome and RNA-seq read data are available for download via the NCBI transcript reads archive and sequence read archive (SRA), respectively (BioProject ID: PRJNA292737).

**Table 1:** Characteristics of the transcriptomic and predicted proteomic datasets for the cercaria and schistosomulum stages of *Trichobilharzia regenti*.

**Sequencing statistics**	
Total raw reads sequenced (cercariae; schistosomula)	226,839,830 (102,569,960; 124,269,870)
Total reads trimmed (cercariae; schistosomula)	146,921,480 (75,733,168; 71,188,312)
Total reads trimmed, normalized (cercariae; schistosomula)	28,859,275 (10,953,728; 17,905,547)
Average read length of trimmed reads (mean ± standard deviation)	132±30 bases
**Transcriptome assemblies**	
Total number of reads for assembly	28,859,275
Contigs of ≥105 nucleotides (mean ± S.D.; range)	20,958 (2007.2±2063.13; 105–41,111)
Number of reads that mapped to contigs (%)	16,337,592 (67,9)
Number of non-redundant proteins of ≥30 amino acids predicted (mean ±S.D.; range)	12,705 (514.5±537.1; 30–8,133)
Complete/partial matches to 248 CEGMA-encoded proteins (%)	87.9/89.5
**Numbers of proteins homologous (BLASTp; E-value cut-off off ≤ 10−**^**0.5**^**) to entries in various databases (01 July 2014) (% of predicted proteome)**	
NCBI	10,900 (85.7)
SwissProt	8,347 (65.6)
MEROPS peptidase	318 (2.5)
MEROPS peptidase inhibitor	260 (2.0)
**Numbers of proteins homologous (BLASTp; E-value cut-off of ≤ 10−**^**0.5**^**) to entries in KEGG databases (% of predicted proteome; number of conserved KEGG protein classes or pathways)**	
KEGG BRITE	5,935 (46.7; 3,275)
KEGG PATHWAY	3,611 (28.4; 1,934)
**Numbers of predicted proteins with conserved domains or GO annotations (% of predicted proteome; number of unique InterProScan domains or GO terms)**	
InterProScan conserved domains	10,585 (83.3; 5,115)
GO terms (number of transcripts)	6,961 (54.8; 1,527)
Biological process	3,946 (31.0; 576)
**Numbers of proteins predicted to be excreted/secreted (% of predicted proteome)**	
Predicted E/S proteins	135 (1.1)

**Fig 2:**
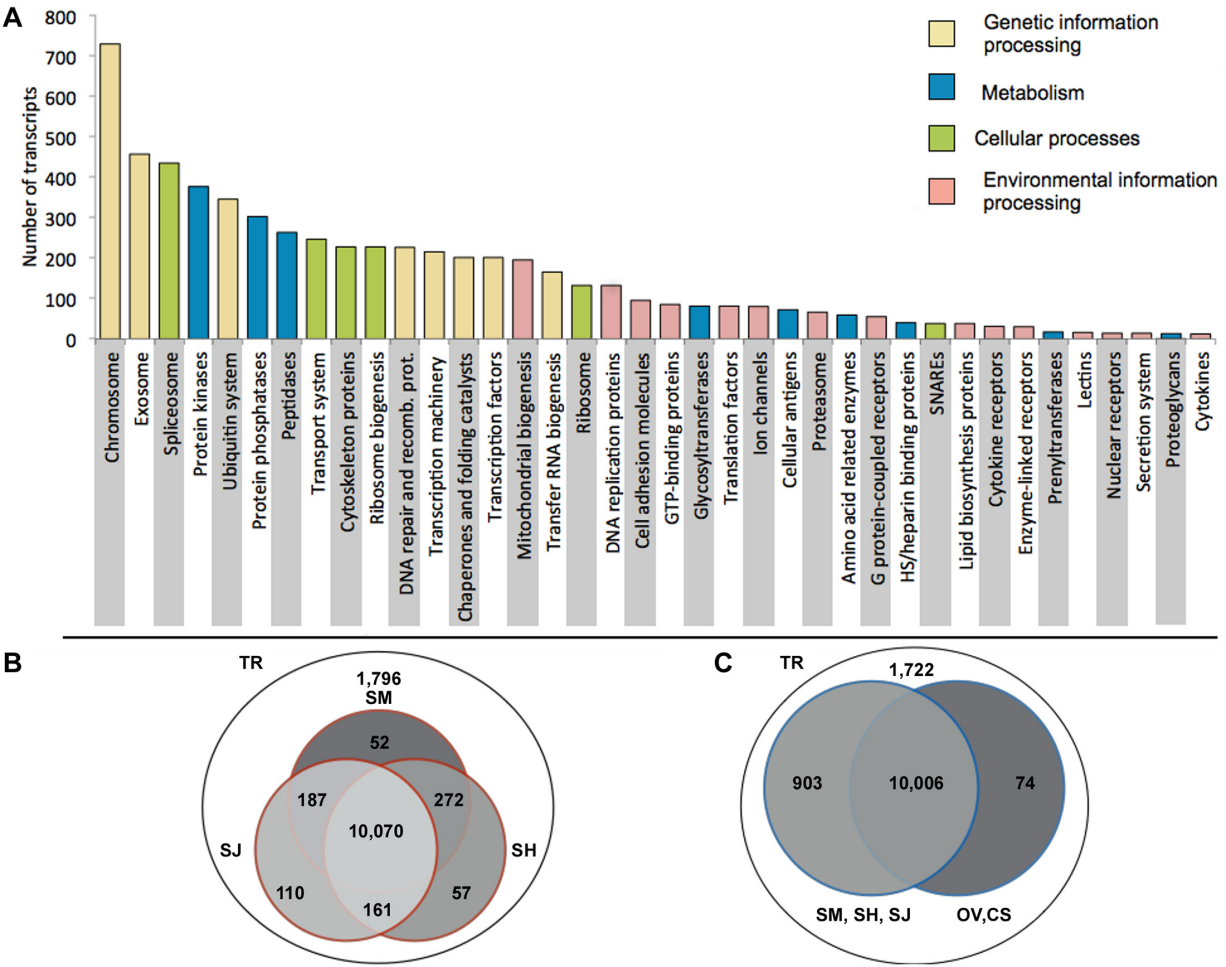
(A) Kyoto Encyclopedia of Genes and Genomes (KEGG) BRITE annotation of transcripts from cercaria and schistosomulum transcriptomes of *Trichobilharzia regenti*. (B, C) Venn diagrams displaying the results from homology-based comparisons of transcripts in the transcriptome of *T*. *regenti* cercariae or schistosomula with the genomes of *Schistosoma mansoni* (SM), *S*. *haematobium* (SH), *S*. *japonicum* (SJ), *Clonorchis sinensis* (CS) and *Opisthorchis viverrini* (OV).

Totals of 88 and 243 potential contaminants (avian, viral and bacterial) were removed from the cercaria and schistosomulum transcriptomes, respectively. Following the removal of these sequences, the larval transcriptome was shown to encode 10,900 (77.5%) and 8,347 (57.8%) predicted proteins that were homologous (BLASTp; E-value ≤ 1e^-05^) to those in the NCBI (non-redundant proteins) and SwissProt databases, respectively (Table 1). In addition, 5,935 predicted proteins were assigned 41 unique KEGG BRITE protein families (Fig 2A; Tables 1 and S2), and 3,611 predicted proteins were assigned 172 biological KEGG pathways (Tables 1 and S2). Using the MEROPS peptidase and inhibitors database, 318 peptidases, including key molecules recognized to be involved in cercarial penetration and schistosomulum migration, nutritional uptake and/or immune evasion [19] were identified (S3 Table). Transcripts encoding metallopeptidases (*n* = 129) were abundant, and included ubiquinol-cytochrome *c* reductase proteins (*n* = 23), kell blood-group proteins (*n* = 8) and leucine aminopeptidases (*n* = 4). Transcripts encoding cysteine peptidases (*n* = 106) including cathepsin B (*n* = 11), cathepsin L (*n* = 6) cathepsin C (= dipeptidyl-peptidase; *n* = 3), ubiquitin-specific peptidases (*n* = 10) and legumains/aspariginyl endopeptidases (*n* = 2) and a ubiquitin-specific peptidase, were also well represented. Transcripts encoding serine peptidases (*n* = 62) represented indeterminate peptidases (*n* = 43), cathepsin A (= carboxypeptidase A; *n* = 3) and mitochondrial inner membrane peptidase 2 (*n* = 2). Transcripts encoding threonine peptidases (*n* = 15) were represented by proteasome subunits (*n* = 4). Also identified was a small number of transcripts encoding aspartic proteases (*n =* 6), including one cathepsin D. In total, transcripts representing 260 inhibitors of peptidases were identified in the (non-redundant) transcriptome of *T*. *regenti*, most of which represented inhibitors of metallo- (*n* = 110), serine (*n* = 77) and cysteine peptidases (*n* = 41) (S3 Table).

A relatively large number of proteins predicted were homologous to those encoded in the genomes of other trematodes, including schistosomes (10,909 proteins; 85.6% being similar to *Schistosoma mansoni*, *S*. *japonicum* and/or *S*. *haematobium*) (Fig 2B) and Asian liver flukes (10,080 proteins; 79.3% being similar to *Clonorchis sinensis* and/or *Opisthorchis viverrini*) (Fig 2C). Of note were 1,722 transcripts that had no homology to other trematode species; only 12 of them (including collagen alpha-3(VI) chain-like, 40S ribosomal protein S7-like, ReO_6, membrane magnesium transporter 1-B-like, VWFA and cache domain containing protein 1 and UPF0729 protein C18orf32 homolog) shared homology to proteins in the NCBI database. In addition, collagen, type VI, alpha and small subunit ribosomal protein S7e were predicted to be involved in five biological pathways (S4 Table). In addition, the larval transcriptome encoded 135 ES proteins, including various peptidases and their inhibitors, phosphatases, kinases, transferases and ribonucleases (S5 Table).

### Differentially transcribed genes, and biological pathways enriched in either cercariae or schistosomula

**Fig 3:**
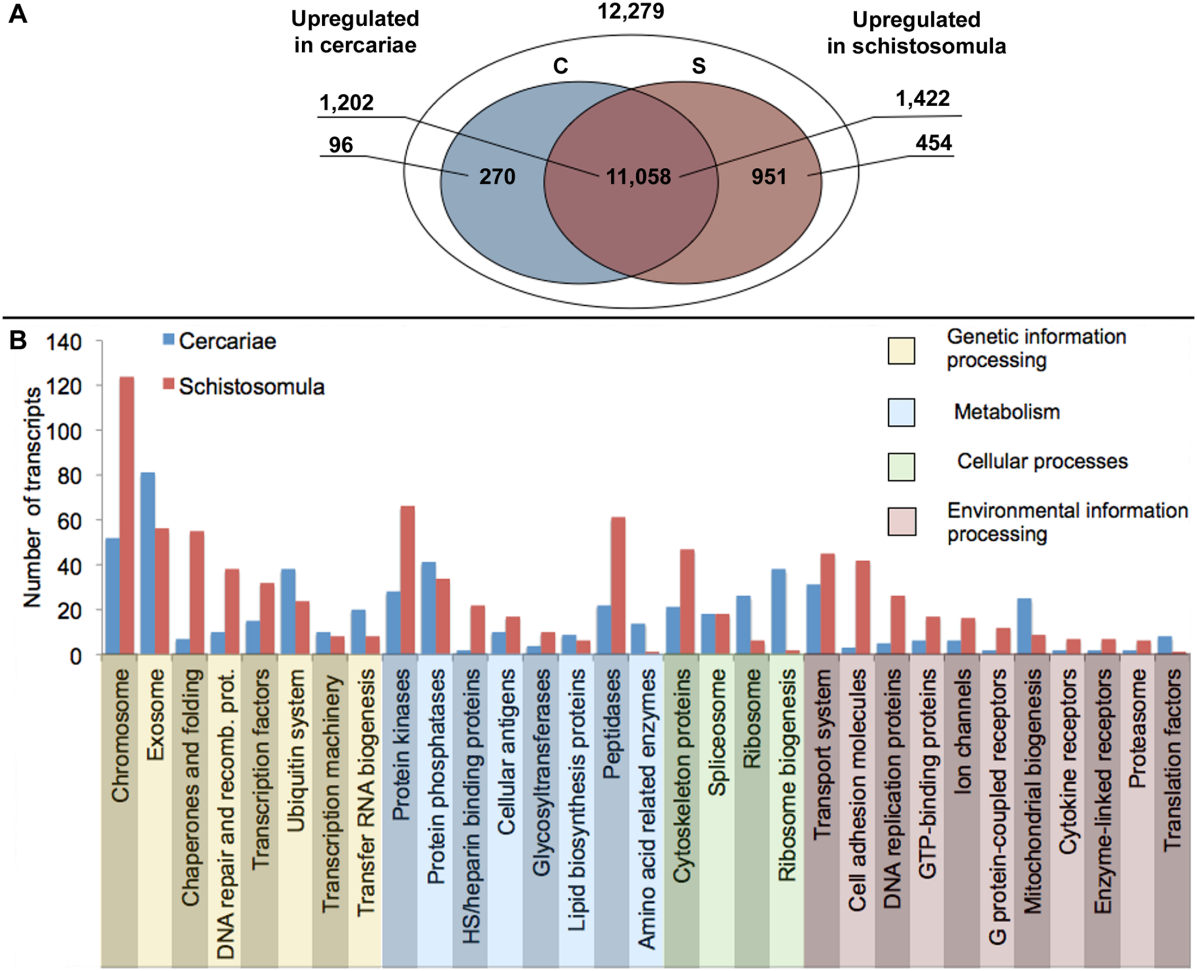
(A) Transcripts expressed exclusively in cercariae, schistosomula or in both developmental stages, based on expected counts derived from RNA-Seq by Expectation Maximization (RSEM) analysis. Cercariae (C) and schistosomula (S); heparan sulphate (HS). (B) Bar graph showing genes differentially transcribed between cercariae and schistosomula of *Trichobilharzia regenti* (Kyoto Encyclopedia of Genes and Genomes (KEGG) BRITE annotation).

A total of 11,058 transcripts were shared by cercariae and schistosomula. Mapping results revealed 270 and 951 transcripts to be specific to the cercaria and schistosomulum stages, respectively (Fig 3A; S6 and S7 Tables). In total, 1,301 transcripts were up-regulated in cercariae and 1,876 in schistosomula (S8 and S9 Tables). The top twenty most differentially transcribed genes in cercariae encoded venom allergen-like protein 8, tegumental protein, calcium-binding proteins, glutamine synthetase and some uncharacterized proteins (with no homology to any sequences in public databases). The top twenty most differentially transcribed genes in schistosomula encoded peptidases (e.g. peptidase M26, cathepsin B1) and beta galactosidase, some uncharacterized proteins with homology to those of *S*. *mansoni*, two of which (Treg_015087, Treg_015334) were homologous to a saposin-like protein and beta hexosaminidase B based on InterPro classification respectively (Table 2). A number of up-regulated transcripts encoded proteins with conserved functional domains (1,178 and 1,699 for cercariae and schistosomula, respectively) and/or similarity to proteins in the KEGG database [605 and 786 (BRITE) (Fig 3B), and 389 and 487 (pathway) for cercariae and schistosomula, respectively]. Using these transcripts, we were able to identify enriched KEGG BRITE protein families, biological pathways and GO terms (biological process) in each of the two larval stages (Tables 3, 4, S10 and S11).

**Table 2:** Twenty most differentially transcribed genes of *Trichobilharzia regenti* cercariae and schistosomula.

Transcript ID	Expected counts	Log2 fold change	NCBI annotation
**Cercariae**			
Treg_000002	33,582	18.4	NO HIT
Treg_000452	27,851	18.3	gi|159792922|gb|ABW98681.1| venom allergen-like protein 8 (*Schistosoma mansoni*)
Treg_000248	20,225	17	gi|256052720|ref|XP_002569901.1| tegumental protein (*S*. *mansoni*)
Treg_003587	19,928	17.1	NO HIT
Treg_003763	10,904	16.5	gi|256075345|ref|XP_002573980.1| Calcium-binding protein 2 (CaBP2) (*S*. *mansoni*)
Treg_000203	10,417	15.9	NO HIT
Treg_000153	10,336	15.4	gi|454247|emb|CAA82847.1| zinc finger protein (putative) (*S*. *mansoni*)
Treg_000941	7,094	15.3	NO HIT
Treg_000009	5,956	15.3	gi|256084831|ref|XP_002578629.1| hypothetical protein (*S*. *mansoni*)
Treg_000069	5,112	15.2	NO HIT
Treg_000112	3,731	15.1	gi|256074546|ref|XP_002573585.1| glutamine synthetase bacteria (*S*. *mansoni*)
Treg_003585	2,948	15.1	gi|256084831|ref|XP_002578629.1| hypothetical protein (*S*. *mansoni*)
Treg_000514	2,425	14.5	gi|256076875|ref|XP_002574734.1| Calmodulin (CaM) (*S*. *mansoni*)
Treg_000442	2,264	14.4	NO HIT
Treg_000878	1,116	14.2	NO HIT
Treg_003936	1,060	14.1	NO HIT
Treg_000031	868.185	14.0	gi|226485052|emb|CAX79803.1| Calcium-binding EF-hand,domain-containing protein (*Schistosoma japonicum*)
Treg_000619	731.25	13.9	NO HIT
Treg_000633	666.5	13.8	gi|256076881|ref|XP_002574737.1| calmodulin (*S*. *mansoni*)
Treg_000137	650	13.8	NO HIT
**Schistosomula**			
Treg_007215	7,386	16.5	NO HIT
Treg_015087	6,081	15.0	gi|353232479|emb|CCD79834.1| hypothetical protein Smp_194910 (*Schistosoma mansoni*); IPR011001|Saposin-like
Treg_006410	5,681	14.9	NO HIT
Treg_006851	3,719	14.3	gi|489236049|ref|WP_003144328.1| peptidase M26 (*Gemella haemolysans*)
Treg_006064	3,587	14.2	gi|350855260|emb|CAZ35935.2| MEG-4 (10.3) family (*S*. *mansoni*)
Treg_014548	2,670	14.2	NO HIT
Treg_015086	2,608	13.8	NO HIT
Treg_020247	1,984	13.5	gi|55793949|gb|AAV65885.1| cathepsin B1 isotype 5 precursor (*Trichobilharzia regenti*)
Treg_015412	1,771	13.3	gi|555955168|ref|XP_005889743.1| PREDICTED: beta-galactosidase-1-like protein isoform X2 (*Bos mutus*)
Treg_006816	1,537	13.3	NO HIT
Treg_015334	1,509	12.8	gi|56757485|gb|AAW26910.1| SJCHGC06873 protein *(Schistosoma japonicum*); IPR025705|Beta-hexosaminidase subunit alpha/beta
Treg_015853	1,500	12.8	gi|195729971|gb|ACG50796.1| cathepsin B1 (*Trichobilharzia szidat*i)
Treg_008400	1,327	12.7	NO HIT
Treg_009334	1,323	12.7	gi|256078798|ref|XP_002575681.1| hypothetical protein (*Schistosoma mansoni*)
Treg_006699	1,082	12.6	gi|353231322|emb|CCD77740.1| MEG-8 family (*S*. *mansoni*)
Treg_006885	975	12.5	gi|226471160|emb|CAX70661.1| Saposin B domain-containing protein (*S*. *japonicum*)
Treg_008899	877	12.5	gi|4099279|gb|AAD00565.1| precursor anti-coagulant SAP-1 (*Schistosoma haematobium*)
Treg_007673	876	12.5	gi|226467490|emb|CAX69621.1| DM9 domain-containing protein (*S*. *japonicum*)
Treg_007426	747	12.4	NO HIT
Treg_006736	677	12.4	NO HIT

**Table 3:** Summary of enriched metabolic pathways and enzyme classes of cercaria stage (C) with comparison to schistosomulum (S) of *Trichobilharzia regenti* based on KEGG BRITE/pathway classification and Gene ontology (GO).

**KEGG pathway/brite classification**			
	No. of up-regulated transcripts (C/S)	No. of EC terms (C/S)	Total (C+S) No. of transcripts/EC terms
***Metabolism***			
KEGG pathways			
carbohydrate metabolism	50/16	35/12	180/87
amino acid metabolism	26/7	16/5	75/43
energy metabolism	25/3	23/3	92/67
metabolism of cofactors and vitamins	15/2	8/2	31/11
nucleotide metabolism;Purine metabolism	24/13	14/11	141/68
KEGG protein class			
lipid biosynthesis proteins	9/6	5/6	38/22
amino acid related enzymes	14/1	7/1	59/29
***Genetic information***			
KEGG pathways			
translation	42/3	38/3	183/128
KEGG protein class			
exosome	81/47	63/38	456/190
***Enviromental information processing***			
KEGG pathways			
signal transduction	21/7	11/6	97/40
***Cellular processes***			
KEGG pathways			
cell growth and death	23/12	11/9	97/38
transport and catabolism	10/2	6/2	26/11
KEGG protein class			
ribosome	26/1	24/1	132/100
ribosome biogenesis	38/2	33/2	227/156
**Categories of biological processes of gene ontology (GO)**			
	Number of transcripts		
Cellular respiration	115		
Carbohydrate metabolism	40		
Glucose metabolism	29		
Actomyosin structure organization	23		
Generation of precursor metabolites and energy	23		
Coenzyme metabolism	22		
Cofactor metabolism	22		
Pyridine-containing compound metabolism	9		

**Table 4:** Summary of enriched metabolic pathways and enzyme classes of schistosomulum stage (S) with comparison to cercaria (C) of *Trichobilharzia regenti* based on KEGG BRITE/pathway classification and Gene ontology (GO).

**KEGG pathway/brite classification**			
	No. of up-regulated transcripts (S/C)	No. of EC terms (S/C)	Total (C+S) No. of transcripts/EC terms
***Metabolism***			
KEGG pathways			
glycan biosynthesis and metabolism	34/2	4/1	54/10
carbohydrate metabolism;Amino sugar and nucleotide sugar metabolism	32/7	3/4	73/23
KEGG protein class			
peptidases	61/22	31/14	263/135
heparan sulfate/heparin binding proteins	22/2	11/2	40/19
proteoglycans	6/0	6/0	13/9
***Genetic information***			
KEGG protein class			
chaperones and folding catalysts	55/7	12/7	20192
***Enviromental information processes***			
KEGG pathways			
signaling molecules and interaction	13/0	12/0	23/14
KEGG protein class			
cell adhesion molecules and their ligands	42/3	28/2	95/47
***Cellular processes***			
KEGG pathways			
lysosome	78/4	20/4	147/51
cell cycle	34/10	26/8	143/76
**Categories of biological processes of gene ontology (GO)**			
		Number of transcripts	
Proteolysis		215	
Cell adhesion		105	
Biological adhesion		37	
Developmental process		20	
Multicellular organismal process		15	

In cercariae, 162 up-regulated transcripts represented five enriched KEGG BRITE protein families (Tables 3 and S10), with most transcripts encoding exosome-related proteins, including calmodulin (*n* = 10), long-chain acyl-CoA synthetase (*n* = 4), creatine kinase (*n* = 4) and tropomyosin 1 (*n* = 3). Other protein families were associated with: (a) metabolic processes involving lipid biosynthesis proteins (*n* = 9) and amino acid-related enzymes (*n* = 14), and (b) cellular processes linked to protein translation, ribosomes (*n* = 26) and ribosome biogenesis (*n* = 38). In addition, 202 transcripts up-regulated in cercariae represented 21 enriched KEGG (biological) pathways (Tables 3 and S10), with almost one quarter of them (*n* = 50) linked to pathways associated with carbohydrate metabolism, including the tricarboxylic acid cycle (*n* = 23), glycolysis (*n* = 21) and pyruvate metabolism (*n* = 13). Other pathways enriched in the cercariae were linked to amino acid metabolism [alanine, aspartate and glutamate (*n* = 11); arginine and proline metabolism (*n* = 11); and glycine, serine and threonine metabolism (*n* = 7)]; energy metabolism [oxidative phosphorylation; *n* = 21; nitrogen metabolism (*n* = 4)]; the metabolism of cofactors and vitamins and nucleotide metabolism [nicotinate and nicotinamide metabolism (*n* = 15) and purine metabolism (*n* = 24)]; environmental information processing [calcium signaling pathway (*n* = 23) and two component regulatory system (*n* = 4)] (Tables 3 and S10). In addition to these enriched protein families and biological pathways, 161 transcripts up-regulated in the cercariae were linked to 36 GO terms for ‘biological process’ that were enriched in the cercarial stage (Tables 3 and S10). These GO terms were represented by eight representative biological processes, including cellular respiration (115 transcripts linked to 17 GO terms, including ATP metabolic process, glycerol-3-phosphate metabolic process, nucleotide biosynthetic process, organophosphate biosynthetic process and oxidation-reduction process), glucose metabolism (29 transcripts representing five GO terms, including carbohydrate catabolic process, cellular carbohydrate metabolic process, glucose metabolic process and organic substance catabolic process), acto-myosin structure organization (23 transcripts representing five GO terms, including actomyosin structural organization, cell wall macromolecule metabolic process, cell wall organization or biogenesis, energy-coupled proton transport, down electrochemical gradient, and ribosome biogenesis) and coenzyme metabolism (22 transcripts representing four GO terms, including acetyl-CoA metabolic process, coenzyme catabolic process, coenzyme metabolic process and cofactor catabolic process). Other biological processes were represented by single GO terms and included carbohydrate metabolism (40 transcripts), cofactor metabolism (*n* = 22), generation of precursor metabolites and energy (*n* = 23) and pyridine-containing compound metabolism (*n* = 9).

In schistosomula, 145 up-regulated transcripts represented five enriched KEGG BRITE protein families (Tables 4 and S11), with most encoding peptidases (61 transcripts) including cathepsin B (*n* = 11), cathepsin D (*n* = 8), separase (*n* = 4), leucyl amino peptidase (*n* = 3) and legumain (*n* = 3). Other protein families enriched were associated with: (a) metabolic processes involving heparan sulfate/heparin binding proteins (*n* = 22) and proteoglycans (*n* = 6), (b) genetic information processing involving chaperones and folding catalysts (*n* = 54) and (c) environmental information processes represented by cell adhesion molecules and their ligands (*n* = 42). In addition, 128 up-regulated transcripts represented 10 enriched KEGG biological pathways (Tables 4 and S11). Metabolic pathways involved carbohydrate, amino sugar or nucleotide sugar metabolism (*n* = 32); glycan biosynthesis and metabolism, including glycosaminoglycan degradation (*n* = 32), and glycosphingolipid biosynthesis (*n* = 33). In each metabolic pathway, 30 different transcripts encoding hexosaminidase were inferred to be involved. Pathways associated with signalling were also significantly enriched in the schistosomula, and represented by cell adhesion molecules (*n* = 6) and extracellular matrix—receptor interaction molecules (*n* = 10). Other enriched pathways in the schistosomula were associated with cellular processes, such as cell cycle (*n* = 21) and processes linked to lysosome function (78 transcripts, including 30 encoding hexosaminidases). In addition to the enriched protein families and biological pathways, 332 transcripts were linked to 28 GO (‘biological process’) terms enriched in the schistosomulum stage (Tables 4 and S11). These GO terms represented five core processes: cell adhesion (*n* = 105 transcripts; 18 associated GO terms), proteolysis (*n* = 215; 7 GO terms), biological adhesion (*n* = 37; one GO term), multicellular organismal process (*n* = 15; one GO term) and developmental processes (*n* = 20; one GO term).

## Discussion

To date, genomic and transcriptomic studies of schistosomatids have focused on *S*. *mansoni*, *S*. *japonicum* and *S*. *haematobium* [52–54]. In the present study, we characterized the first transcriptome of any avian fluke, *T*. *regenti*, undertook comparative studies with other schistosomatids and elucidated differences between two key developmental stages responsible for host invasion (cercaria) and migration through the neural tissues (schistosomulum) at the molecular level.

### Comparison of *T*. *regenti* with other schistosomatids

Based on similarity searches, the proteins predicted from the *T*. *regenti* transcriptome have 85.9% sequence homology to those of human schistosomes (BLASTx, E-value 10^−5^), although divergent molecules (14.2%) are proposed to relate to considerable differences in biology of bird and human schistosomes. *Trichobilharzia regenti* shares 110 transcripts exclusively with *S*. *japonicum*, with no homologues in either *S*. *mansoni* or *S*. *haematobium*. This finding is consistent with *S*. *japonicum* being the most similar of the three human schistosomes to *T*. *regenti* in terms of chemical tools serving for skin penetration. In particular, a shared feature of *T*. *regenti* and *S*. *japonicum* is the absence, at both the mRNA and protein levels, of cercarial elastase [18,55–57]. By contrast, *S*. *mansoni* and *S*. *haematobium* both use elastase for cercarial invasion of the definitive host [58,59]. While humans represent exclusive/dominant hosts of *S*. *haematobium* and *S*. *mansoni*, *S*. *japonicum* has an ability to infect a broader range of mammals (including pigs, water buffalo and water rats; [60]), but not birds as natural final hosts. In this case, therefore, the absence of elastase in both *S*. *japonicum* and *T*. *regenti* seems to be an interesting example of convergence. In other words, the data here indicate that *T*. *regenti* shares more unique transcripts (n = 110) with *S*. *japonicum* than either *S*. *mansoni* (n = 52) or *S*. *haematobium* (n = 57) (Fig 2B); the apparent absence of cercarial elastase from the two former species might reflect their distinct host affiliations/broader spectra compared with the latter two. Interestingly, 1,722 (13.6%) of predicted proteins of *T*. *regenti* had no homology to those of any other trematode species, for which molecular data are available (none of which are parasites of birds) (Fig 2B). Of these 1,722 predicted proteins, only 151 had homologues in public databases. Remaining 1,571 transcripts represented orphans (genes with no homology to known domains or proteins). However, some of these orphans were highly transcribed in cercariae and/or schistosomula. Interestingly, 10 and 8 of 20 genes exhibiting the highest transcription in cercariae and schistosomula, respectively, encoded orphans. Such molecules might have unique particular biological functions or processes in *T*. *regenti* associated with the penetration of avian skin (cercaria), specific migration through neural tissue (schistosomulum) and an adaptation to the avian definitive host (both stages). This statement is supported by biological and clinical evidence [26], showing that *T*. *regenti* is not able to effectively establish infection in the mammalian hosts under natural conditions.

### Molecular aspects unique to the cercarial stage of *T*. *regenti*

In digenetic trematodes, interestingly, the cercaria are considered to be less transcriptionally active than other developmental stages [61–63], although the high transcription of genes encoding proteins participating in particular metabolic pathways described here likely reflects the specific biological requirements of this larval stage.

#### Carbohydrate metabolism

After leaving their snail intermediate host, schistosome cercariae must find and penetrate the epidermis of their definitive host within hours [16], and thus rely exclusively on stored energy reserves (including glycogen) [16]. In *T*. *regenti* cercariae, the enriched carbohydrate metabolism, including glycolysis (linked to glucose-6-phosphatase, fructose-1,6-bisphosphatase, phosphoenolpyruvate carboxykinase and pyruvate carboxylase), pyruvate, citrate cycle and oxidative phosphorylation pathways indicate the importance of aerobic degradation of glucose from glycogen sources, consistent with observations in other free-living trematode stages [64–68]. However, interestingly, other pathways for the degradation of fructose, mannose, pentose phosphate, starch, sucrose, galactose, glyoxylate and dicarboxylate were also enriched in *T*. *regenti* cercariae and appear to be functional; the present results indicate that some enzymes likely participate in multiple metabolic pathways. For instance, citrate synthase is likely involved in glyoxylate and dicarboxylate metabolism as well as in the citrate cycle, the latter of which is regarded as a major metabolic pathway in *T*. *regenti* cercariae, corresponding to evidence for *S*. *mansoni* [69].

#### Translation and amino acid metabolism

The present findings show that *T*. *regenti* cercariae are less transcriptionally active than schistosomula, although the pathways linked to translational machinery, such as ribosome biogenesis, were significantly enriched, including 24 distinct large ribosomal subunit proteins. We speculate that cercariae synthesize ribosomes for immediate protein synthesis following their invasion of the definitive host, in which the energy and protein sources would appear to be unlimited. The limited pool of amino acids in schistosome cercariae might originate from the snail intermediate host and/or from biosynthesis [70]; in *T*. *regenti*, the transcripts encoding enzymes involved in the metabolism of amino acids, such as alanine (alanine transaminase), asparagine (aspartate-ammonia ligase), glutamine (glutamine synthetase) and cysteine (cystathionine gamma-lyase), were identified. All of these amino acids are derived from the citrate cycle or glycolysis, considered the main metabolic pathways in the cercarial stage. Although the synthesis of amino acids by parasitic stages of helminths is well recognized [71], this aspect has been seldom studied in free-living stages of schistosomes [62,72].

#### Key role for calcium during invasion

In *T*. *regenti* cercariae, calcium signalling may also be important for enriched cellular processes, as has been observed for *S*. *japonicum* [62]. For example, transcripts encoding calcium-binding protein (CaBP) represented the fifth most differentially transcribed gene (log-fold change: 16.5) between cercariae and schistosomula of *T*. *regenti* (cf. Table 2), which likely relates to CaBP being essential during the very active invasion process of larvae in the definitive host [73]. In other schistosomes, such as *S*. *mansoni*, there is a down regulation of CaBP in cercariae following epidermal penetration [73]. The preacetabular (circumacetabular) penetration glands of schistosome cercariae contain a high concentration of calcium [18,74,75]. This calcium has been suggested to regulate the activity of *S*. *mansoni* cercarial elastase [76,77] and of glycocalyx shedding by cross-linking endogenous postacetabular mucopolysaccharides [18,76]. Although there is no evidence here of elastase in the *T*. *regenti* cercaria, the highest transcription of any peptidase in this stage was exhibited by the calpain gene, which is strictly regulated by calcium ions. Calpain may regulate the surface membrane synthesis process, as it does in *S*. *mansoni* [78], but this proposals needs to be tested.

### Molecular processes unique to the schistosomulum stage of *T*. *regenti*

During their invasion of the definitive host, the transformation of cercariae to schistosomula is associated with rapid and major morphological, biochemical and molecular changes. Unlike most schistosomes studied to date, the schistosomula of *T*. *regenti* do not enter the bloodstream, but rather seek out and migrate in nerves and consume neural tissue (as nutrition) during migration [26]. Compared with the circulatory system, the nervous system represents an immunologically and physiologically distinct environment (in terms of nutrients and immune responses). The present study investigated the schistosomula of *T*. *regenti* seven days after infection of the avian host and explored the molecular adaptations required for the parasite to establish and survive in this unique niche—the neural system.

#### Growth and development, and cell adhesion molecules (CAMs)

In addition to playing critical roles in regulating normal cell integrity and cell-cell interactions, cell adhesion molecules (CAMs) can also regulate host-parasite interactions. For example, in protozoan parasites, CAMs have been reported to mediate the attachment of pathogens to host cells [79]. Whilst CAMs of *T*. *regenti* might participate in host-parasite interactions, it seems more likely that they regulate cell adhesion to maintain their multicellular structure [80]. Highly up-regulated transcription associated with CAMs in the schistosomula of *T*. *regenti* might indicate rapid growth and development of different organ structures within the definitive host. For instance, abundant transcription linked to neuroligin, netrin receptor and semaphorin might relate to the development of the nervous system in this developmental stage, based on evidence from other organisms [81,82].

#### Metabolism

Contrary to the situation in the cercariae of *T*. *regenti*, transcription of genes encoding enzymes involved in aerobic metabolic processes in the schistosomula is very low, and limited to the citrate cycle and oxidative phosphorylation. This finding suggests that the schistosomulum possesses a microaerobic metabolism seven days after infection of the definitive host, which is similar to observations made for human schistosomes [25]. However, in the schistosomulum stage of *S*. *mansoni*, for example, most energy is reported to originate via anaerobic glycolysis, with lactate as an end product [24,25,83], contrasting the situation in the *T*. *regenti* schistosomulum.

#### Proteolysis and histolysis during migration

The schistosomula of *T*. *regenti* need to migrate through the avian neural tissues to reach the nasal mucosa, where they mature to adults, mate and produce eggs [1]. The most abundant transcripts specifically in the schistosomulum stage encode proteolytic enzymes, including several cysteine peptidases, in particular, transcripts encoding cathepsin B1.5 (Treg_006320) (RSEM-expected counts: 30,779), followed by cathepsin L (Treg_006337) (12,349), cathepsin B1.6 (Treg_004279) (8,216), cathepsin L-like peptidase (Treg_006792) (5,514), cathepsin C (Treg_014726) (5,145) and leucine amino peptidase (4,966) (Treg_015017) (S9 Table). While cercariae store and express proteolytic enzymes in penetration glands for invasion via the skin, schistosomula degrade various host tissues during migration [84], including neural tissue, specifically in the case of *T*. *regenti* [26], and evade or block host immune attack [75]. The present study indicates that *T*. *regenti* schistosomula employ a considerably broader arsenal of proteolytic enzymes than cercariae do, with more than twice as many peptidases (n = 31) predicted to be upregulated in the former than in the latter stages, respectively.

According to a previous study [85], the digestive peptidase cathepsin B1 of *T*. *regenti* schistosomula (TrCB1) represents at least 6 distinct isoforms (TrCB1.1 to TrCB1.6). The isoforms TrCB1.5 and TrCB1.6 have the catalytic cysteine substituted by glycin and are thus inactive, and were previously reported to be the least abundant members of all six known isoforms [85]. Surprisingly, the present transcriptomic data indicate that the two genes encoding TrCB1.5 and TrCB1.6 have the highest transcription of any cathepsin B detected here in *T*. *regenti*. In metazoan organisms, the existence of inactive enzyme forms (also of peptidases) is quite common, and the percentage of expression of inactive/active isoforms usually ranges from 10% to 15%; inactive paralogs of peptidases are speculated to have a regulatory function, achieved by a competition with active peptidase forms for their substrate(s) [86]. Also present among the highly transcribed peptidases was cathepsin B2 (TrCB2; RSEM-expected counts: 4,386). Cathepsins B2 as well as B1 were previously identified as dominant peptidases produced by *T*. *regenti* schistosomula, with a major capacity to degrade myelin basic protein, one of the principal components of spinal cord tissues, whereas haemoglobinolytic activity was negligible [20,85,87]. These findings may explain the ability of schistosomula to migrate through the white matter of the spinal cord, composed predominantly of neuronal axons, and cause axonal damage (cf. [26]).

Abundant transcription (RSEM-expected counts: 12,349) in *T*. *regenti* was also linked to cathepsin L. This enzyme has broad specificity in various parasitic flatworms, and can assume various functions. Importantly, it can cleave a range of substrates, including collagen, laminin, fibronectin, haemoglobin and immunoglobulins [88–90], and is likely intimately involved in the migration of *T*. *regenti* schistosomula and their ability to modulate or evade host immune attack. The immunomodulatory effect of proteolytic enzymes has been investigated in other schistosomes, and the aspartic peptidase, cathepsin D, is recognized as a key representative that cleaves host IgG from the tegumental surface of adult worms [91]. Besides this immunomodulatory role, cathepsin D is known to participate in the primary cleavage of haemoglobin [92]. In the present study, cathepsin D was represented by nine highly up-regulated transcripts in the schistosomula of *T*. *regenti*. As schistosomula of this species do not feed on blood, this enzyme in this developmental stage might assume an imunomodulatory role, but this hypothesis needs to be tested. In summary, the high level of transcription associated with cathepsins in the schistosomulum of *T*. *regenti* provides further support for the significance of this group of enzymes in parasitic flatworms. In addition to cathepsins, schistosomula also transcribe genes encoding a broad array of proteolytic enzymes that participate in numerous biological processes and likely have key roles during the parasite’s invasion, survival and longevity in the definitive host.

#### Lysosomal proteins

In addition to the cysteine peptidases discussed, a variety of other proteins linked to lipid processing, including lysophospholipase III, Niemann-Pick C2 protein, palmitoyl-protein thioesterase and saposin-like proteins (SAPs), were encoded in the transcriptome representing the schistosomulum stage of *T*. *regenti*. Palmitoyl-protein thioesterase is also encoded in the genomes of human schistosomes and *C*. *sinensis* [52–54,93] and is likely involved in the degradation of lipoproteins [94]. In relation to lipid trafficking, one transcript encoding a lipid-binding Niemann-Pick C2 protein, associated with cholesterol trafficking from the lysosome [95], was transcribed exclusively in *T*. *regenti* schistosomula. Genes encoding homologous proteins have been identified in the genomes of other trematodes, such as *S*. *mansoni*, *S*. *japonicum*, *S*. *haematobium*, *C*. *sinensis* and *O*. *viverrini* [52–54,93,96]. In addition, 10 different transcripts encoding SAPs were identified; SAPs can play diverse functional roles often through their interaction with lipids, lipid-degrading enzymes and lipid-presenting molecules [97], which may involve the activation of lipid-degrading enzymes, such as sphingolipid glycohydrolase [98]. Alternatively, SAPs might directly distrupt lipid membranes via the formation of pore-forming structures [99]. These proteins have been reported for trematodes including *S*. *mansoni* and *Fasciola* spp. and are presumed to facilitate the degradation of ingested host cells [100,101]. The high transcription of genes coding for proteins that interact with or bind to lipids might associate with the molecular machinery that *T*. *regenti* uses to degrade and then digest lipid-rich neural tissue, likely being critical for parasite migration and nutrition.

Interestingly, 38 different transcripts were predicted to encode hexosaminidase B, 30 were up-regulated in the schistosomula and 20 were uniquely transcribed in this stage. The analysis of enriched protein classes and pathways encoded by genes in schistosomula was biased by these different transcripts, which represent multiple KEGG classes, such that our interpretation is guarded. Although the function of hexosaminidase B in *T*. *regenti* is presently enigmatic, this enzyme is encoded in the genomes of human schistosomes [52–54] and *O*. *viverrini* [96]. We suspect that the high number of transcripts encoding this enzyme in *T*. *regenti* schistosomula might relate to multiple functions in this developmental stage. Based on independent evidence from humans, hexosaminidase can degrade sphingolipids (gangliosides) in neural tissue [102], and mutation of the hexosaminidase genes can lead to lethal neurodegenerative disorders (Tay-Sachs and Sandhoff or lysosomal storage disease) which are caused by an accumulation of gangliosides in the nervous system [103,104]. We suggest that an overexpression of hexosaminidase in *T*. *regenti* schistosomula leads to a degradation of neural tissue during their migration. In humans, only the alpha-subunit of hexosaminidase B is known to be involved in lipid degradation. As a corresponding alpha-subunit appears not to be encoded in *T*. *regenti*, the role of the hexosaminidase B should be explored. Although very little is known about hexosaminidases of helminths, homologs appear to have one or more roles in parasite-host interactions, for example, in the parasitic nematode *Trichinella spiralis* due to its specific sugar-binding property (lectin-like activity) or its specific glycohydrolase catalytic activity [105].

In conclusion, we describe here the first molecular exploration of neuropathogenic *Trichobilharzia* of birds and identify key biological pathways and proteins central to the invasion of the avian host and migration and development within neural tissues. Of particular significance is that we have been able to: (i) dissect the molecular differences between the cercarial stage (during cutaneous penetration) and the schistosomula stage (during neural migration and establishment within the avian host) and (ii) establish some biological distinctions between neuropathogenic *T*. *regenti* of birds and related blood flukes (schistosomes) of humans. The present annotated transcriptome for *T*. *regenti* provides a useful resource for comparative studies of schistosomatids and other trematodes, and should also underpin future genomic and proteomic investigations of *T*. *regenti*.

## Supporting Information

S1 Table Summary of raw reads, trimmed reads, trimmed and normalized reads for the transcriptomic data of cercariae and schistosomula of *Trichobilharzia regenti*. Results of mapping of paired, trimmed, corrected, non-normalized reads from cercariae and schistosomula to the final non-redundant transcriptome of *Trichobilharzia regent*i. P1—pair read 1; P2—pair read 2; STD—Standard deviation
(XLSX)

S2 Table Summary of the classification of predicted proteins from transcriptome of cercariae and schistosomula of *Trichobilharzia regenti* based on homology (BLASTp; *E*-value ≤ 10^−5^) to annotated proteins in the Kyoto Encyclopedia of Genes and Genomes (KEGG) BRITE functional hierarchies database, and pathway maps for cellular and organismal functions. (XLSX)

S3 Table Summary of peptidases and their inhibitors encoded in the transcriptome representing *Trichobilharzia regenti*, classified into families and subfamilies, with type enzyme listed in the MEROPS peptidase database. UP—Unassigned peptidase
(XLSX)

S4 Table Orphan transcripts of the combined cercaria/schistosomulum transcriptome of Trichobilharzia regenti with no homology to other trematodes and classified based on NCBI and Kyoto Encyclopedia of Genes and Genomes (KEGG) databases. (XLSX)

S5 Table Transcripts of *Trichobilharzia regenti* predicted to encode excretory/secretory (ES) proteins, annotated using the National Center for Biotechnology Information (NCBI) database. Transcription level established based on RNA-Seq by Expectation Maximization (RSEM) analysis. Sorted in descending order based on “expected counts” for the schistosomula stage. Differential transcripts are marked with an asterisk.
(XLSX)

S6 Table Summary of transcripts uniquely transcribed in each cercaria and schistosomulum of *Trichobilharzia regenti* based on read mapping to the (consensus) transcriptome. Annotation using the National Center for Biotechnology Information (NCBI) database, and level of transcription based on RNA-Seq by Expectation Maximization (RSEM) analysis.
(XLSX)

S7 Table Transcripts uniquely transcribed in either the cercaria or schistosomulum of *Trichobilharzia regenti*, classified using the Kyoto Encyclopedia of Genes and Genomes (KEGG) database. (XLSX)

S8 Table Summary of transcripts upregulated in the cercaria stage of *Trichobilharzia regenti* based on read mapping to the (consensus) transcriptome with National Center for Biotechnology Information (NCBI) annotation. Sorted in descending based on log2-fold change of differential transcription.
(XLSX)

S9 Table Summary of transcripts upregulated in the schistosomulum stage of *Trichobilharzia regenti* based on read mapping to (consensus) transcriptome, using National Center for Biotechnology Information (NCBI) annotation. Sorted in descending order based on log2-fold change of differential transcription.
(XLSX)

S10 Table Summary of transcripts enriched in the cercaria stage of *Trichobilharzia regenti* based on Kyoto Encyclopedia of Genes and Genomes (KEGG) BRITE/pathway and gene ontology (GO) classifications. Transcripts uniquely transcribed in the cercaria are marked with an asterisk.
(XLSX)

S11 Table Summary of transcripts enriched in the schistosomulum stage of *Trichobilharzia regenti* based on Kyoto Encyclopedia of Genes and Genomes (KEGG) BRITE/pathway and gene ontology (GO) classifications. Transcripts uniquely transcribed in the schistosomulum are marked with an asterisk.
(XLSX)

## References

[1] Horak P, Kolarova L, Dvorak J. Trichobilharzia regenti n. sp. (Schistosomatidae, Bilharziellinae), a new nasal schistosome from Europe. Parasite. 1998;5: 349–357. 9879557 10.1051/parasite/1998054349

[2] Horak P, Kolarova L, Adema CM. Biology of the schistosome genus *Trichobilharzia*. Adv Parasitol. 2002;52: 155–233. 12521261 10.1016/s0065-308x(02)52012-1

[3] Jouet D, Skirnisson K, Kolarova L, Ferte H. Molecular diversity of Trichobilharzia franki in two intermediate hosts (Radix auricularia and Radix peregra): a complex of species. Infect Genet Evol. 2010;10: 1218–1227. 10.1016/j.meegid.2010.08.001 20708105

[4] Korsunenko A V, Chrisanfova GG, Ryskov AP, Movsessian SO, Vasilyev VA, Semyenova SK. Detection of European Trichobilharzia schistosomes (T. franki, T. szidati, and T. regenti) based on novel genome sequences. J Parasitol. 2010;96: 802–806. 10.1645/GE-2297.1 20677938

[5] Davis NE. Identification of an avian schistosome recovered from Aythya novaeseelandia and infectivity of its miracidia to Lymnaea tomentosa snails. J Helminthol. 2006;80: 225–233. 16923264

[6] Gohardehi S, Fakhar M, Madjidaei M. Avian schistosomes and human cercarial dermatitis in a wildlife refuge in Mazandaran Province, northern Iran. Zoonoses Public Health. 2013;60: 442–447. 10.1111/zph.12020 23121919

[7] Horak P, Dvorak J, Kolarova L, Trefil L. *Trichobilharzia regenti*, a pathogen of the avian and mammalian central nervous systems. Parasitology. 1999;119 (Pt 6): 577–581. 10633919 10.1017/s0031182099005132

[8] Hradkova K, Horak P. Neurotropic behaviour of *Trichobilharzia regenti* in ducks and mice. J Helminthol. 2002;76: 137–141. 10.1079/JOH2002113 12015826

[9] Chanová M, Bulantová J, Máslo P, Horák P. In vitro cultivation of early schistosomula of nasal and visceral bird schistosomes (Trichobilharzia spp., Schistosomatidae). Parasitol Res. 2009;104: 1445–52. 10.1007/s00436-009-1343-y 19238442

[10] Horák P, Kolářová L. Snails, waterfowl and cercarial dermatitis. Freshw Biol. Blackwell Publishing Ltd; 2011;56: 779–790. 10.1111/j.1365-2427.2010.02545.x

[11] Soldanova M, Selbach C, Kalbe M, Kostadinova A, Sures B. Swimmer’s itch: etiology, impact, and risk factors in Europe. Trends Parasitol. 2013;29: 65–74. 10.1016/j.pt.2012.12.002 23305618

[12] Horak P, Mikes L, Lichtenbergova L, Skala V, Soldanova M, Brant SV. Avian schistosomes and outbreaks of cercarial dermatitis. Clin Microbiol Rev. 2015;28: 165–190. 10.1128/CMR.00043-14 25567226 PMC4284296

[13] Jelinek, Nothdurft, Loscher. Schistosomiasis in Travelers and Expatriates. J Travel Med. 1996;3: 160–164. 9815445 10.1111/j.1708-8305.1996.tb00731.x

[14] Kourilová P, Kolárová L. Variations in immunofluorescent antibody response against *Trichobilharzia* and Schistosoma antigens in compatible and incompatible hosts. Parasitol Res. 2002;88: 513–21. 10.1007/s00436-002-0607-6 12107473

[15] Neuhaus W. [Biology and development of Trichobilharzia Szidati N. Sp. (Trematoda, Schistosmatidae), a parasite causing dermatitis in man]. Z Parasitenkd. Not Available; 1952;15: 203–266. 14951656 10.1007/BF00260453

[16] Lawson JR, Wilson R a. The survival of the cercariae of Schistosoma mansoni in relation to water temperature and glycogen utilization Parasitology. 1980 pp. 337–348. 10.1017/S0031182000056079 7443297

[17] Ligasova A, Bulantova J, Sebesta O, Kasny M, Koberna K, Mikes L. Secretory glands in cercaria of the neuropathogenic schistosome Trichobilharzia regenti—ultrastructural characterization, 3-D modelling, volume and pH estimations. Parasit Vectors. 2011;4: 162 10.1186/1756-3305-4-162 21854564 PMC3171358

[18] Mikes L, Zìdková L, Kasný M, Dvorák J, Horák P. In vitro stimulation of penetration gland emptying by Trichobilharzia szidati and T. regenti (Schistosomatidae) cercariae. Quantitative collection and partial characterization of the products. Parasitol Res. 2005;96: 230–41. 10.1007/s00436-005-1347-1 15868186

[19] Kasny M, Mikes L, Hampl V, Dvorak J, Caffrey CR, Dalton JP, et al Chapter 4. Peptidases of trematodes Advances in parasitology. 2009 pp. 205–297. 10.1016/S0065-308X(09)69004-7 19622410

[20] Doleckova K, Kasny M, Mikes L, Cartwright J, Jedelsky P, Schneider EL, et al The functional expression and characterisation of a cysteine peptidase from the invasive stage of the neuropathogenic schistosome *Trichobilharzia regenti*. Int J Parasitol. 2009;39: 201–211. 10.1016/j.ijpara.2008.06.010 18708063 PMC2625449

[21] Horak P, Kovar L, Kolarova L, Nebesarova J. Cercaria-schistosomulum surface transformation of *Trichobilharzia szidati* and its putative immunological impact. Parasitology. 1998;116 (Pt 2): 139–147. 9509023 10.1017/s0031182097002059

[22] Mclaren DJ, Hockley DJ. Blood flukes have a double outer membrane. Nature.; 1977;269: 147–149. 71658 10.1038/269147a0

[23] Horemans AM, Tielens AG, van den Bergh SG. The reversible effect of glucose on the energy metabolism of *Schistosoma mansoni* cercariae and schistosomula. Mol Biochem Parasitol. 1992;51: 73–79. 1565140 10.1016/0166-6851(92)90202-u

[24] Skelly PJ, Stein LD, Shoemaker CB. Expression of *Schistosoma mansoni* genes involved in anaerobic and oxidative glucose metabolism during the cercaria to adult transformation. Mol Biochem Parasitol. 1993;60: 93–104. 10.1016/0166-6851(93)90032-S 8396206

[25] Parker-Manuel SJ, Ivens AC, Dillon GP, Wilson RA. Gene expression patterns in larval Schistosoma mansoni associated with infection of the mammalian host. PLoS Negl Trop Dis. 2011;5: e1274 10.1371/journal.pntd.0001274 21912711 PMC3166049

[26] Lichtenbergova L, Lassmann H, Jones MK, Kolarova L, Horak P. *Trichobilharzia regenti*: host immune response in the pathogenesis of neuroinfection in mice. Exp Parasitol. 2011;128: 328–335. 10.1016/j.exppara.2011.04.006 21554878

[27] Chanova M, Horak P. Terminal phase of bird schistosomiasis caused by Trichobilharzia regenti (Schistosomatidae) in ducks (Anas platyrhynchos f. domestica). Folia Parasitol (Praha). 2007;54: 105–107. 17886739

[28] Kolarova L, Horak P, Cada F. Histopathology of CNS and nasal infections caused by *Trichobilharzia regenti* in vertebrates. Parasitol Res. 2001;87: 644–650. 11511002 10.1007/s004360100431

[29] Bolger AM, Lohse M, Usadel B. Trimmomatic: a flexible trimmer for Illumina sequence data. Bioinformatics. 2014;30: 2114–2120. 10.1093/bioinformatics/btu170 24695404 PMC4103590

[30] FastQC. A quality control tool for high throughput sequence data. Babraham Bioinforma Web site http//www.bioinformatics.babraham.ac.uk/projects/fastqc/.

[31] Nurk S, Bankevich A, Antipov D, Gurevich AA, Korobeynikov A, Lapidus A, et al Assembling single-cell genomes and mini-metagenomes from chimeric MDA products. J Comput Biol. 2013;20: 714–737. 10.1089/cmb.2013.0084 24093227 PMC3791033

[32] Brown CT, Howe A, Zhang Q, Pyrkosz AB, Brom TH, Lansing E, et al A Reference-Free Algorithm for Computational Normalization arXiv : 1203. 4802v2 [q-bio. GN] 21 5 2012: 1–18.

[33] Schulz MH, Zerbino DR, Vingron M, Birney E. Oases: robust de novo RNA-seq assembly across the dynamic range of expression levels. Bioinformatics. 2012;28: 1086–1092. 10.1093/bioinformatics/bts094 22368243 PMC3324515

[34] Fu L, Niu B, Zhu Z, Wu S, Li W. CD-HIT: accelerated for clustering the next-generation sequencing data Bioinformatics. 2012 pp. 3150–3152. 10.1093/bioinformatics/bts565 PMC3516142 23060610

[35] Haas BJ, Papanicolaou A, Yassour M, Grabherr M, Blood PD, Bowden J, et al De novo transcript sequence reconstruction from RNA-seq using the Trinity platform for reference generation and analysis. Nat Protoc. 2013;8: 1494–512. 10.1038/nprot.2013.084 23845962 PMC3875132

[36] Parra G, Bradnam K, Korf I. CEGMA: a pipeline to accurately annotate core genes in eukaryotic genomes. Bioinformatics. 2007;23: 1061–1067. 10.1093/bioinformatics/btm071 17332020

[37] Database resources of the National Center for Biotechnology Information. Nucleic Acids Res. 2014;42: D7–17. 10.1093/nar/gkt1146 24259429 PMC3965057

[38] Smit AFA, Hubley R GP. RepeatMasker Open-3.0. [http://www.repeatmasker.org] webcite.

[39] Schwarz EM, Korhonen PK, Campbell BE, Young ND, Jex AR, Jabbar A, et al The genome and developmental transcriptome of the strongylid nematode *Haemonchus contortus*. Genome Biol. 2013;14: R89 10.1186/gb-2013-14-8-r89 23985341 PMC4053716

[40] Magrane M, Consortium U. UniProt Knowledgebase: a hub of integrated protein data. Database (Oxford). 2011;2011: bar009 10.1093/database/bar009 21447597 PMC3070428

[41] Rawlings ND, Barrett AJ, Bateman A. MEROPS: the peptidase database. Nucleic Acids Res. 2010;38: D227–33. 10.1093/nar/gkp971 19892822 PMC2808883

[42] Kanehisa M, Goto S. KEGG: kyoto encyclopedia of genes and genomes. Nucleic Acids Res. 2000;28: 27–30. 10592173 10.1093/nar/28.1.27 PMC102409

[43] Zdobnov EM, Apweiler R. InterProScan—an integration platform for the signature-recognition methods in InterPro. Bioinformatics. 2001;17: 847–848. 11590104 10.1093/bioinformatics/17.9.847

[44] Supek F, Bosnjak M, Skunca N, Smuc T. REVIGO summarizes and visualizes long lists of gene ontology terms. PLoS One. 2011;6: e21800 10.1371/journal.pone.0021800 21789182 PMC3138752

[45] Blum T, Briesemeister S, Kohlbacher O. MultiLoc2: integrating phylogeny and Gene Ontology terms improves subcellular protein localization prediction. BMC Bioinformatics. 2009;10: 274 10.1186/1471-2105-10-274 19723330 PMC2745392

[46] Li B, Dewey CN. RSEM: accurate transcript quantification from RNA-Seq data with or without a reference genome. BMC Bioinformatics. 2011;12: 323 10.1186/1471-2105-12-323 21816040 PMC3163565

[47] Robinson MD, McCarthy DJ, Smyth GK. edgeR: a Bioconductor package for differential expression analysis of digital gene expression data. Bioinformatics. 2010;26: 139–140. 10.1093/bioinformatics/btp616 19910308 PMC2796818

[48] R Development Core Team. R: A Language and Environment for Statistical Computing [Internet]. Vienna, Austria; 2008 Available: http://www.r-project.org

[49] Risso D, Schwartz K, Sherlock G, Dudoit S. GC-content normalization for RNA-Seq data. BMC Bioinformatics. 2011;12: 480 10.1186/1471-2105-12-480 22177264 PMC3315510

[50] Dillies M-A, Rau A, Aubert J, Hennequet-Antier C, Jeanmougin M, Servant N, et al A comprehensive evaluation of normalization methods for Illumina high-throughput RNA sequencing data analysis. Brief Bioinform. 2013;14: 671–683. 10.1093/bib/bbs046 22988256

[51] Alexa A and Rahnenfuhrer J. topGO: Enrichment analysis for Gene Ontology. R package version 2.20.0. [Internet]. 2010. Available: http://www.bioconductor.org/packages/release/bioc/html/topGO.html

[52] Young ND, Jex AR, Li B, Liu S, Yang L, Xiong Z, et al Whole-genome sequence of *Schistosoma haematobium*. Nat Genet. 2012;44: 221–225. 10.1038/ng.1065 22246508

[53] Berriman M, Haas BJ, LoVerde PT, Wilson RA, Dillon GP, Cerqueira GC, et al The genome of the blood fluke *Schistosoma mansoni*. Nature. 2009;460: 352–358. 10.1038/nature08160 19606141 PMC2756445

[54] The Schistosoma japonicum genome reveals features of host-parasite interplay. Nature. Macmillan Publishers Limited. All rights reserved; 2009;460: 345–351. Available: 10.1038/nature08140 PMC3747554 19606140

[55] Kasny M, Mikes L, Dalton JP, Mountford AP, Horak P. Comparison of cysteine peptidase activities in *Trichobilharzia regenti* and *Schistosoma mansoni* cercariae. Parasitology. 2007;134: 1599–1609. 10.1017/S0031182007002910 17517170

[56] Doleckova K, Kasny M, Mikes L, Mutapi F, Stack C, Mountford AP, et al Peptidases of Trichobilharzia regenti (Schistosomatidae) and its molluscan host Radix peregra S. Lat. (Lymnaeidae): construction and screening of cDNA library from intramolluscan stages of the parasite. Folia Parasitol (Praha). 2007;54: 94–98. 17886737

[57] Dvorák J, Mashiyama ST, Braschi S, Sajid M, Knudsen GM, Hansell E, et al Differential use of protease families for invasion by schistosome cercariae. Biochimie. 2008;90: 345–58. 10.1016/j.biochi.2007.08.013 17936488

[58] Curwen RS, Wilson RA. Invasion of skin by schistosome cercariae: some neglected facts. Trends Parasitol. 2003;19: 63–68. 12586470 10.1016/s1471-4922(02)00019-3

[59] Salter JP, Choe Y, Albrecht H, Franklin C, Lim K-C, Craik CS, et al Cercarial elastase is encoded by a functionally conserved gene family across multiple species of schistosomes. J Biol Chem. 2002;277: 24618–24624. 10.1074/jbc.M202364200 11986325

[60] Gray DJ, Williams GM, Li Y, Chen H, Forsyth S, Li R, et al The role of bovines in human *Schistosoma japonicum* infection in the Peoples’ Republic of China. Am J Trop Med Hyg. 2009;81: 301–301.

[61] Jolly ER, Chin C-S, Miller S, Bahgat MM, Lim KC, DeRisi J, et al Gene expression patterns during adaptation of a helminth parasite to different environmental niches. Genome Biol. 2007;8: R65 10.1186/gb-2007-8-4-r65 17456242 PMC1896014

[62] Gobert GN, Moertel L, Brindley PJ, McManus DP. Developmental gene expression profiles of the human pathogen *Schistosoma japonicum*. BMC Genomics. 2009;10: 128 10.1186/1471-2164-10-128 19320991 PMC2670322

[63] Verjovski-Almeida S, DeMarco R, Martins E a L, Guimarães PEM, Ojopi EPB, Paquola ACM, et al Transcriptome analysis of the acoelomate human parasite *Schistosoma mansoni*. Nat Genet. 2003;35: 148–157. 10.1038/ng1237 12973350

[64] Tielens AG, van den Heuvel JM, van den Bergh SG. The energy metabolism of *Fasciola hepatica* during its development in the final host. Mol Biochem Parasitol. 1984;13: 301–307. 6527693 10.1016/0166-6851(84)90121-x

[65] Boyunaga H, Schmitz MG, Brouwers JF, Van Hellemond JJ, Tielens AG. *Fasciola hepatica* miracidia are dependent on respiration and endogenous glycogen degradation for their energy generation. Parasitology. 2001;122: 169–173. 11272647 10.1017/s0031182001007211

[66] Prosdocimi F, Faria-Campos AC, Peixoto FC, Pena SDJSDJ, Ortega JMJM, Franco GRGR. Clustering of *Schistosoma mansoni* mRNA sequences and analysis of the most transcribed genes: Implications in metabolism and biology of different developmental stages. Mem Inst Oswaldo Cruz. 2002;97: 61–69. 10.1590/S0074-02762002000900014 12426597

[67] Tielens AG, Van der Meer P, van den Heuvel JM, van den Bergh SG. The enigmatic presence of all gluconeogenic enzymes in *Schistosoma mansoni* adults. Parasitology. 1991;102 Pt 2: 267–276. 1649428 10.1017/s0031182000062582

[68] Burenina EA. [Properties of gluconeogenesis enzymes from flatworms]. Zh Evol Biokhim Fiziol. 2001;37: 85–91. 11452789

[69] Van Oordt BE, Tielens AG, Van den Bergh SG. Aerobic to anaerobic transition in the carbohydrate metabolism of *Schistosoma mansoni* cercariae during transformation in vitro. Parasitology. 1989;98 Pt 3: 409–415. 2771447 10.1017/s0031182000061497

[70] Tielens A. G. M., Hellemond van JJ. Unusual aspects of metabolism in flatworm parasites Parasitic flatworms: molecular biology, biochemistry, immunology and physiology. CAB International; 2006 10.1079/9780851990279.0387

[71] Barrett J. Amino acid metabolism in helminths. Adv Parasitol. ENGLAND; 1991;30: 39–105. 10.1016/s0065-308x(08)60306-1 2069074

[72] Santos TM, Johnston D a, Azevedo V, Ridgers IL, Martinez MF, Marotta GB, et al Analysis of the gene expression profile of *Schistosoma mansoni* cercariae using the expressed sequence tag approach. Mol Biochem Parasitol. 1999;103: 79–97. 10514083 10.1016/s0166-6851(99)00100-0

[73] Ram D, Grossman Z, Markovics A, Avivi A, Ziv E, Lantner F, et al Rapid changes in the expression of a gene encoding a calcium-binding protein in *Schistosoma mansoni*. Mol Biochem Parasitol. 1989;34: 167–175. 2710168 10.1016/0166-6851(89)90008-x

[74] Dorsey CH, Stirewalt MA. Schistosoma mansoni: localization of calcium-detecting reagents in electron-lucent areas of specific preacetabular gland granules. Z Parasitenkd. 1977;54: 165–173. 605648 10.1007/BF00380799

[75] Modha J, Redman CA, Thornhill JA, Kusel JR. Schistosomes: unanswered questions on the basic biology of the host-parasite relationship. Parasitol Today. 1998;14: 396–401. 17040829 10.1016/s0169-4758(98)01321-0

[76] Dresden MH, Edlin EM. *Schistosoma mansoni*: effect of some cations on the proteolytic enzymes of cercariae. Exp Parasitol. 1974;35: 299–303. 4206936 10.1016/0014-4894(74)90036-8

[77] McKerrow JH, Jones P, Sage H, Pino-Heiss S. Proteinases from invasive larvae of the trematode parasite *Schistosoma mansoni* degrade connective-tissue and basement-membrane macromolecules. Biochem J. 1985;231: 47–51. 3904737 10.1042/bj2310047 PMC1152701

[78] Siddiqui AA, Zhou Y, Podesta RB, Karcz SR, Tognon CE, Strejan GH, et al Characterization of Ca(2+)-dependent neutral protease (calpain) from human blood flukes, Schistosoma mansoni. Biochim Biophys Acta. 1993;1181: 37–44. 8457603 10.1016/0925-4439(93)90087-h

[79] Rowe JA, Claessens A, Corrigan RA, Arman M. Adhesion of *Plasmodium falciparum*-infected erythrocytes to human cells: molecular mechanisms and therapeutic implications. Expert Rev Mol Med. 2009;11: e16 10.1017/S1462399409001082 19467172 PMC2878476

[80] Gumbiner BM. Cell adhesion: the molecular basis of tissue architecture and morphogenesis. Cell. 1996;84: 345–357. 8608588 10.1016/s0092-8674(00)81279-9

[81] Larrivee B, Freitas C, Suchting S, Brunet I, Eichmann A. Guidance of vascular development: lessons from the nervous system. Circ Res. 2009;104: 428–441. 10.1161/CIRCRESAHA.108.188144 19246687

[82] Ou C-Y, Shen K. Setting up presynaptic structures at specific positions. Curr Opin Neurobiol. 2010;20: 489–493. 10.1016/j.conb.2010.04.011 20471244 PMC3168548

[83] Skelly PJ, Shoemaker CB. Induction cues for tegument formation during the transformation of *Schistosoma mansoni* cercariae. Int J Parasitol. 2000;30: 625–631. 10779576 10.1016/s0020-7519(00)00031-x

[84] Caffrey CR, McKerrow JH, Salter JP, Sajid M. Blood “n” guts: an update on schistosome digestive peptidases. Trends Parasitol. 2004;20: 241–8. 10.1016/j.pt.2004.03.004 15105025

[85] Dvorak J, Delcroix M, Rossi A, Vopalensky V, Pospisek M, Sedinova M, et al Multiple cathepsin B isoforms in schistosomula of *Trichobilharzia regenti*: identification, characterisation and putative role in migration and nutrition. Int J Parasitol. 2005;35: 895–910. 10.1016/j.ijpara.2005.02.018 15950230

[86] Pils B, Schultz J. Inactive enzyme-homologues find new function in regulatory processes. J Mol Biol. 2004;340: 399–404. 10.1016/j.jmb.2004.04.063 15210342

[87] Dolecková K, Albrecht T, Mikes L, Horák P. Cathepsins B1 and B2 in the neuropathogenic schistosome *Trichobilharzia regenti*: distinct gene expression profiles and presumptive roles throughout the life cycle. Parasitol Res. 2010;107: 751–5. 10.1007/s00436-010-1943-6 20556428

[88] Berasaín P, Goñi F, McGonigle S, Dowd a, Dalton JP, Frangione B, et al Proteinases secreted by *Fasciola hepatica* degrade extracellular matrix and basement membrane components. J Parasitol. 1997;83: 1–5. Available: http://www.ncbi.nlm.nih.gov/pubmed/9057688 9057688

[89] Collins PR, Stack CM, O’Neill SM, Doyle S, Ryan T, Brennan GP, et al Cathepsin L1, the major protease involved in liver fluke (Fasciola hepatica) virulence: propetide cleavage sites and autoactivation of the zymogen secreted from gastrodermal cells. J Biol Chem. 2004;279: 17038–17046. 10.1074/jbc.M308831200 14754899

[90] Yamakami K, Hamajima F, Akao S, Tadakuma T. Purification and characterization of acid cysteine protease from metacercariae of the mammalian trematode parasite *Paragonimus westermani*. Eur J Biochem. 1995;233: 490–497. 7588793 10.1111/j.1432-1033.1995.490_2.x

[91] Verity CK, Loukas A, McManus DP, Brindley PJ. *Schistosoma japonicum* cathepsin D aspartic protease cleaves human IgG and other serum components. Parasitology. 2001;122: 415–421. 11315174 10.1017/s0031182001007521

[92] Delcroix M, Sajid M, Caffrey CR, Lim K-C, Dvorák J, Hsieh I, et al A multienzyme network functions in intestinal protein digestion by a platyhelminth parasite. J Biol Chem. 2006;281: 39316–29. 10.1074/jbc.M607128200 17028179

[93] Wang X, Chen W, Huang Y, Sun J, Men J, Liu H, et al The draft genome of the carcinogenic human liver fluke *Clonorchis sinensis*. Genome Biol. 2011;12: R107 10.1186/gb-2011-12-10-r107 22023798 PMC3333777

[94] Vesa J, Hellsten E, Makela TP, Jarvela I, Airaksinen T, Santavuori P, et al A single PCR marker in strong allelic association with the infantile form of neuronal ceroid lipofuscinosis facilitates reliable prenatal diagnostics and disease carrier identification. Eur J Hum Genet. 1993;1: 125–132. 7914464 10.1159/000472399

[95] Chikh K, Vey S, Simonot C, Vanier MT, Millat G. Niemann-Pick type C disease: importance of N-glycosylation sites for function and cellular location of the NPC2 protein. Mol Genet Metab. 2004;83: 220–230. 15542393 10.1016/j.ymgme.2004.06.013

[96] Young ND, Nagarajan N, Lin SJ, Korhonen PK, Jex AR, Hall RS, et al The *Opisthorchis viverrini* genome provides insights into life in the bile duct. Nat Commun. 2014;5: 4378 10.1038/ncomms5378 25007141 PMC4104445

[97] Bruhn H. A short guided tour through functional and structural features of saposin-like proteins. Biochem J. 2005;389: 249–257. 10.1042/BJ20050051 15992358 PMC1175101

[98] Furst W, Sandhoff K. Activator proteins and topology of lysosomal sphingolipid catabolism. Biochim Biophys Acta. 1992;1126: 1–16. 1606169 10.1016/0005-2760(92)90210-m

[99] Leippe M, Tannich E, Nickel R, van der Goot G, Pattus F, Horstmann RD, et al Primary and secondary structure of the pore-forming peptide of pathogenic *Entamoeba histolytica*. EMBO J. 1992;11: 3501–3506. 1396552 10.1002/j.1460-2075.1992.tb05432.x PMC556807

[100] Don TA, Bethony JM, Loukas A. Saposin-like proteins are expressed in the gastrodermis of *Schistosoma mansoni* and are immunogenic in natural infections. Int J Infect Dis. 2008;12: e39–47. 10.1016/j.ijid.2007.10.007 18571965

[101] Espino AM, Hillyer G V. Molecular cloning of a member of the *Fasciola hepatica* saposin-like protein family. J Parasitol. 2003;89: 545–552. 10.1645/GE-3113 12880256

[102] Mark BL, Mahuran DJ, Cherney MM, Zhao D, Knapp S, James MNG. Crystal structure of human beta-hexosaminidase B: understanding the molecular basis of Sandhoff and Tay-Sachs disease. J Mol Biol. 2003;327: 1093–1109. 12662933 10.1016/s0022-2836(03)00216-x PMC2910754

[103] Sandhoff K, Harzer K. Gangliosides and gangliosidoses: principles of molecular and metabolic pathogenesis. J Neurosci. 2013;33: 10195–10208. 10.1523/JNEUROSCI.0822-13.2013 23785136 PMC6618597

[104] Mahuran DJ. Biochemical consequences of mutations causing the GM2 gangliosidoses. Biochim Biophys Acta. 1999;1455: 105–138. 10571007 10.1016/s0925-4439(99)00074-5

[105] Rhoads ML. Purification, characterization, and immunochemical studies of beta-N-acetyl-D-hexosaminidase from the parasitic nematode *Trichinella spiralis*. Mol Biochem Parasitol. 1988;31: 57–69. 2972930 10.1016/0166-6851(88)90145-4

